# *RecView*: an interactive R application for locating recombination positions using pedigree data

**DOI:** 10.1186/s12864-023-09807-2

**Published:** 2023-11-25

**Authors:** Hongkai Zhang, Bengt Hansson

**Affiliations:** Department of Biology, Lund University, Lund, 22362 Sweden

**Keywords:** Recombination, Crossover, Non-crossover, R Shiny, Next generation sequencing, SNP

## Abstract

**Background:**

Recombination reshuffles alleles at linked loci, allowing genes to evolve independently and consequently enhancing the efficiency of selection. This makes quantifying recombination along chromosomes an important goal for understanding how selection and drift are acting on genes and chromosomes.

**Results:**

We present *RecView*, an interactive R application and its homonymous R package, to facilitate locating recombination positions along chromosomes or scaffolds using whole-genome genotype data of a three-generation pedigree. *RecView* analyses and plots the grandparent-of-origin of all informative alleles along each chromosome of the offspring in the pedigree, and infers recombination positions with either of two built-in algorithms: one based on change in the proportion of the alleles with specific grandparent-of-origin, and one on the degree of continuity of alleles with the same grandparent-of-origin. *RecView* handles multiple offspring and chromosomes simultaneously, and all putative recombination positions are reported in base pairs together with an estimated precision based on the local density of informative alleles. We demonstrate *RecView* using genotype data of a passerine bird with an available reference genome, the great reed warbler (*Acrocephalus arundinaceus*), and show that recombination events can be located to specific positions.

**Conclusions:**

*RecView* is an easy-to-use and highly effective application for locating recombination positions with high precision. *RecView* is available on GitHub (https://github.com/HKyleZhang/RecView.git).

**Supplementary Information:**

The online version contains supplementary material available at 10.1186/s12864-023-09807-2.

## Introduction

Recombination generates novel genetic variation by creating new haplotypes and combinations of alleles at linked loci. As a result, it enables linked genes to evolve somewhat independently, thereby enhancing the efficiency of selection [1, 2]. When recombination is suppressed, selection acts on large, linked regions, leading to reduced fixation probability of beneficial mutations and lowered efficiency to purge mildly harmful mutations. Indeed, the outcome of selection on any genomic region is dependent on the combined effect on linked loci, which, in turn, is influenced by the rate of recombination. The significance of recombination in maintaining the fitness of extensive genomic regions is exemplified by the rapid degeneration and substantial loss of genes observed on non-recombining regions of sex chromosomes [1–4].

The recombination landscape can be quantified by comparing genetic and physical maps [5–8] and by analysing linkage disequilibrium (LD) along chromosomes, with the rational that high recombination rates lead to faster LD decay [9–11]. Additionally, recombination resulting from single crossover events can be studied by direct observations of crossovers, or chiasmata, using cytological approaches [12, 13]. These methods provide broad-scale patterns of recombination along chromosomes, but do not offer data on the specific positions of single recombination events. To determine precise recombination positions, one can explore genome-wide allele sharing patterns between grandparents and grandchildren. Recent advancements in high-throughput sequencing have made this method available for almost any study species, as long as biological samples over at least three generations can be gathered [14]. The level of resolution in determining recombination positions in such analyses depends on the sequencing method, the extent of genetic variation within the study species, and the quality of the reference genome. Genome-wide approaches, highly heterozygous species, and high-quality assemblies, generally yield higher resolution. By scaling these analyses to include multiple grandchildren and covering numerous meiotic recombination events, it becomes possible to statistically analyse whether specific genomic features drive recombination, explore potential recombinational differences between males and females, as well as estimate population-averaged recombination rates. We anticipate that this progress will increasingly make the identification of recombination positions in pedigree data a common practice in future population genetic and evolutionary studies.

To facilitate locating recombination positions, we developed *RecView*, an interactive R application, designed to infer recombination positions along chromosomes in whole-genome sequence data in a three-generation pedigree. *RecView* requires a genotype file with unphased (bi-allelic) single nucleotide polymorphism (SNP) data of individuals in a three-generation pedigree and a scaffold file providing the order and orientation of the scaffolds on each chromosome. Essentially, the analysed recombination events occur in the parents (F1 generation in the pedigree) and *RecView* analyses the grandparent-of-origin (GoO) of alleles at each SNP in each offspring (F2 generation in the pedigree) separately. The genotypes of the four grandparents, the two parents and the focal offspring are sometimes informative for the GoO inferences. We refer to such cases as informative SNPs. For example, this is true if one of the grandparents carries allele *C* at an SNP-locus, and if *C* is inherited through the pedigree to the offspring (e.g., *AC*_paternal grandfather_-*AA*_paternal grandmother_-*AA*_maternal grandfather_-*AA*_maternal grandmother_-*AC*_father_-*AA*_mother_-*AC*_offspring_). *RecView* evaluates all SNPs and plots the GoO of informative SNPs along each chromosome (or scaffold). The approximate positions of all putative recombination events can be viewed in a chromosome-wide GoO plot, and *RecView* also locates and outputs the putative recombination positions on the chromosomes by applying either of two algorithms that we developed: (i) the proportional difference (PD) algorithm that detects positions where the difference in the proportion of alleles with specific GoO between flanking windows reaches a local maximum, and (ii) the cumulative continuity score (CCS) algorithm that detects positions where the continuous inferences of a specific GoO switch from one grandparent to the other. *RecView* also calculates and reports an estimated precision of each putative recombination position based on the local density of informative alleles. The main results are output as chromosome-wide plots and as tables.

In this study, we introduce *RecView* and demonstrate its applicability using short-read sequence data obtained from two offspring, as well as their grandparents and parents, of the great reed warbler (*Acrocephalus arundinaceus*), a passerine bird with an available reference genome [15]. We show that recombination events can be located to specific positions, and that *RecView* can handle multiple offspring and chromosomes simultaneously. Furthermore, we assess the sensitivity of the analysis by analysing datasets comprising 10% and 1% of the original full data. This provides valuable insights into the impact of SNP density, which can be useful for choosing an appropriate sequencing method, such as whole-genome or reduced representation sequencing.

## Implementation

### Workflow of *RecView*

**Fig. 1:**
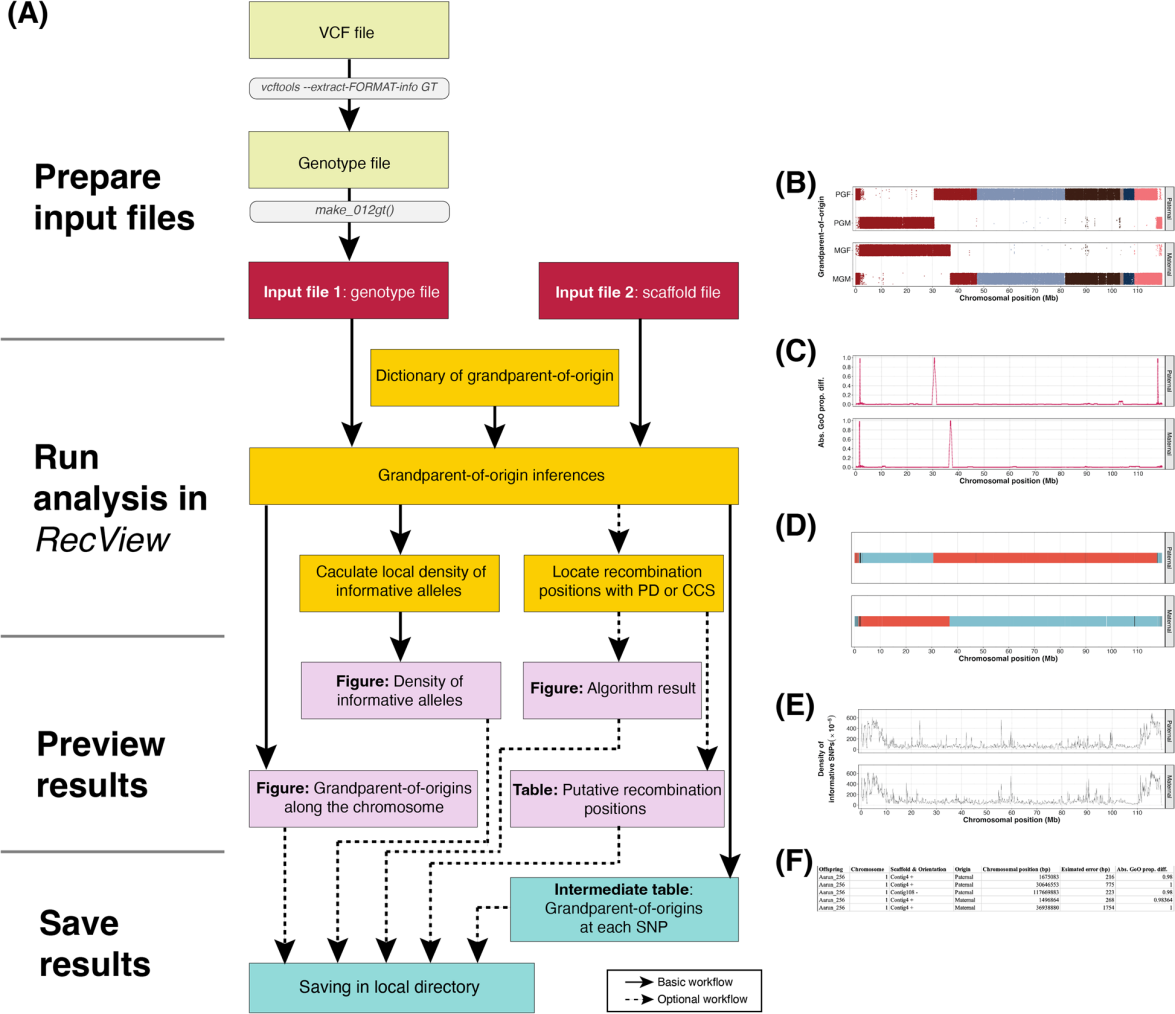
(**A**) The workflow of *RecView*. Solid lines indicate the basic workflow while dashed lines indicate the optional workflow. *RecView* requires an input genotype file which can be generated by using *make_012gt()* on the output file from *VCFtools*. *RecView* further requires an input scaffold file containing the order and orientation of the scaffolds. These two input files are used together with the built-in dictionary of grandparent-of-origin (GoO) to produce (**B**) a GoO figure showing the GoO inferences of alleles along the scaffolds, and (**E**) a figure showing the informative alleles density. *RecView* can further locate putative recombination positions with the proportional difference or cumulative continuity score algorithms and output (**C**,** D**) result figures and (**F**) tables. The result figures and tables can be saved, including an intermediate table containing the GoO inferences at each SNP

**Fig. 2:**
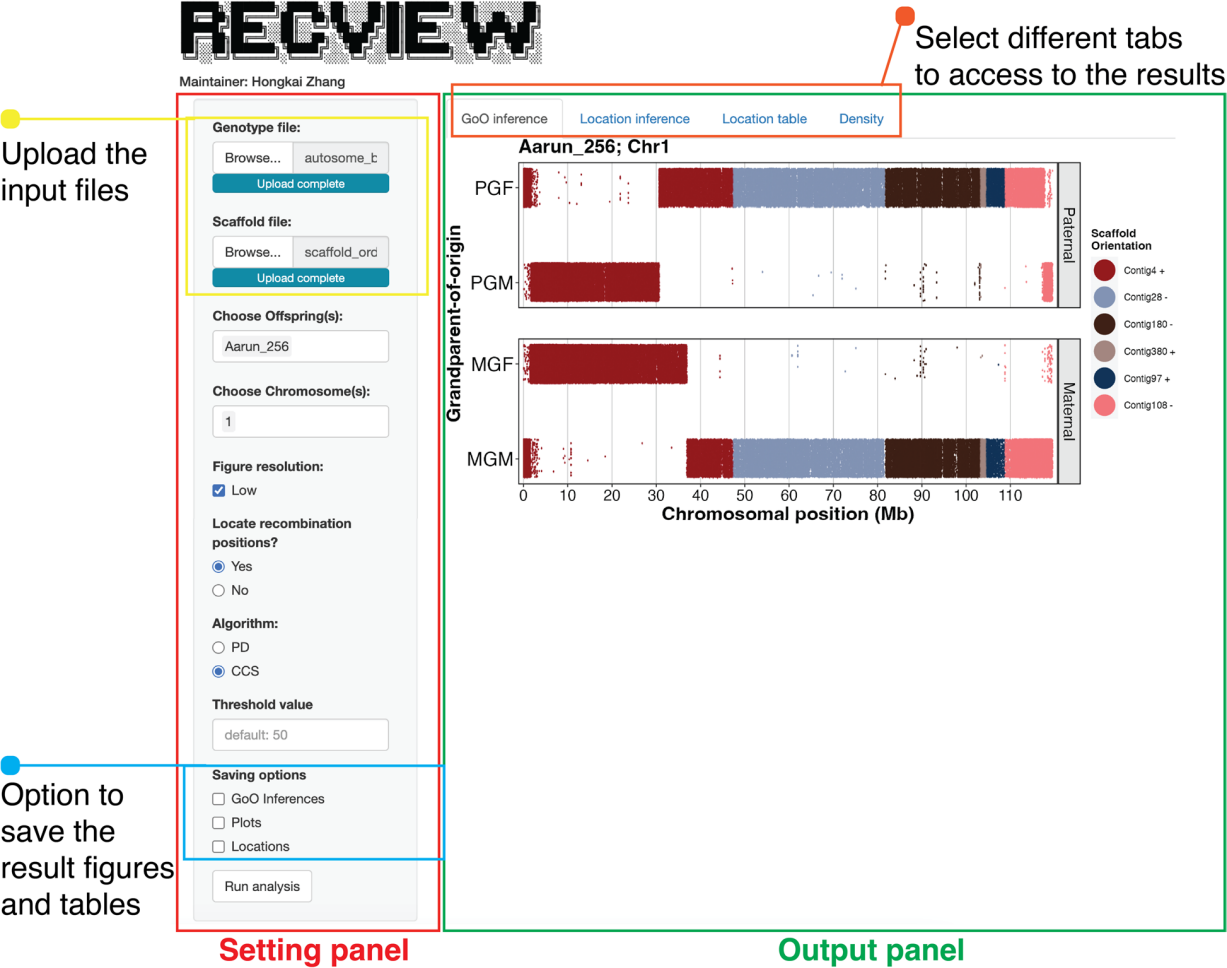
The GUI of *RecView* with the setting panel (red square) for uploading input files (yellow square), setting options, and saving options (blue square), and the output panel (green square) where results can be accessed by selecting different tabs (orange square)

The *RecView* ShinyApp and its homonymous R package are available for download and installation through GitHub (https://github.com/HKyleZhang/RecView.git). *RecView* uses the local machine as server and runs offline. *RecView* is intended to provide an easy-to-use graphic user interface (GUI) to locate recombination positions using pedigree data. The basic workflow of *RecView* is shown in Fig. 1 and the GUI in Fig. 2.

*RecView* requires two input files, a genotype file and a scaffold file. The genotype file consists of unphased biallelic genotypes of the individuals in the pedigree, and we provide a built-in function, *make_012gt()*, to transform genotype output from *VCFtools* into the software-acceptable format (coding genotypes as: 0 = reference allele homozygote; 2 = alternative allele homozygote; 1 = heterozygote). During the analysis of each offspring, the combined genotypes of the pedigree individuals at each SNP form a 7-digit genotype string ordered from grandparents, parents, with males before females, and the offspring. For example, the genotype string 1000101 at an SNP means that the paternal grandfather, the father and the offspring are heterozygote, while the remaining individuals are homozygote for the reference allele. The scaffold file provides the order and orientation of the scaffolds and needs to have five columns with the following headings (case sensitive): “scaffold” (the label of the scaffold; character), “size” (the size of the scaffold in bp; integer), “CHR” (the chromosome the scaffold belongs to; character), “order” (the order of the scaffold on the chromosome; integer), and “orientation” (the scaffold orientation on the chromosome; + or -). Data for each scaffold are given in separate rows.

The ShinyApp GUI is initiated by the command *run_RecView_App()* in the “Viewer” tab in Rstudio. In the GUI, there are options to select (i) the input files from the local folder, (ii) which offspring and chromosomes to be analysed, (iii) the resolution of the screen graphs, (iv) whether to locate recombination positions, (v) whether to use the PD or the CCS algorithm to locate recombination positions, (vi) which parameters and thresholds to use for PD and CCS, and (vii) whether to save the results as plots and tables (Fig. 2).

After choosing options, the analysis is initiated by selecting “*Run analysis*”. The analysis infers the GoO for the alleles at each SNP by searching and matching the specific genotype string (e.g., 1000101) of the pedigree individuals to a “dictionary of GoO” – a list including all possible (e.g., 1000101) genotype strings. Impossible genotype strings (e.g., 0000002) are not in the dictionary. Given that the dictionary of GoO encompasses all possible genotype strings and that numerous SNPs will share identical strings, this search-and-match process provides a highly efficient method for inferring the GoO of a large number of SNPs. Next, depending on selected options, the analysis locates the recombination positions with either the PD or the CCS algorithm. The output includes three result plots and a table: the GoO inferences plot, the plot showing the results of applying PD or CCS, the plot with density of informative alleles, along the chromosome(s), and a table containing information of the putative recombination positions and their estimated precision (Fig. 1).

Details of the GoO analysis, the PD and CCS algorithms, and how the estimated precision for recombination positions are calculated, are given in Supplementary 1. The default parameter settings for PD are a window size of 550 SNPs, a step of 17 SNPs, a finer step of 1 SNP, and a threshold of 0.9, and for the CCS the default threshold is 50. These can be modified depending on, e.g., SNP density.

### Example dataset for demonstrating *RecView* applicability

We demonstrate the applicability of *RecView* using data from two chromosomes (chromosome 1, size 119.6 Mb; chromosome 21, size 9.2 Mb) of a passerine bird species with an available reference genome, the great reed warbler (*Acrocephalus arundinaceus*) [15]. This species has, as most passerines, 39 autosomal chromosomes and a pair of sex chromosomes.

We randomly selected a three-generation pedigree, including 4 grandparents, 2 parents and 2 offspring (ID-256 and ID-258), from our long-term study population of great reed warblers at Lake Kvismaren, southern Central Sweden (59°10ʹ N, 15°24ʹ E; [16–20]). For these 8 individuals, we downloaded raw sequencing reads from the BioProject PRJNA970100 on NCBI [21].

The sequence reads were trimmed with *trimmomatic version 0.39* [22], mapped to the great reed warbler genome assembly [15] using *bwa mem* version 0.7.17 [23], and read duplicates were removed with *PicardTools* version 2.27.5 [24]. Then, a VCF file of called variants were produced with *freebayes* version 1.3.2 [25], and the genotypes at bi-allelic SNPs were extracted with *VCFtools* version 0.1.16 (option: --extract-FORMAT-info GT; [26]). The whole-genome dataset was reduced to contain only chromosome 1 and 21. In addition to this dataset, we downsampled the number of SNPs to 10% and 1% of the original number (referred to as the “10% downsampled dataset” and “1% downsampled dataset”), to assess the sensitivity of the analysis for SNP density and mimic a situation where fewer SNPs are available for the analysis, such as for reduced representation sequencing data (e.g., restriction site-associated (RAD) sequencing data; [20]).

We loaded the *RecView* R package on a local computer, and used the *make_012gt()* function to generate the genotype files (this was done for all three datasets; full, 10% and 1%, respectively). We prepared a scaffold file according to the instructions above, using the ordered and oriented scaffolds of the great reed warbler assembly ([15]; B. Hansson et al., unpubl.). Then, we ran the analyses in *RecView* using the default parameters for PD and CCS given above.

Note, however, that *RecView* can analyse complete genomes (all chromosomes or scaffolds), and multiple offspring, simultaneously.

## Results

### Full dataset

**Fig. 3:**
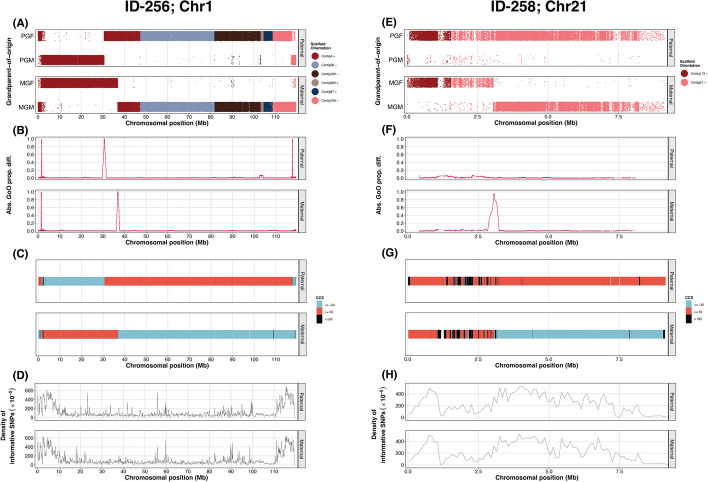
Result plots for chromosome 1 in offspring ID-256 and chromosome 21 in offspring ID-258. (**A**, **E**) The grandparental-of-origin of informative alleles at all SNPs along chromosome 1 in offspring ID-256 (**A**) and chromosome 21 in offspring ID-258 (**E**). Each dot represents an allele at a specific SNP for the paternal or maternal chromosomes. Dots are plotted with noise on the y-axis to alleviate the degree of overlap. Colouration indicates different scaffolds on the chromosomes in the great reed warbler genome assembly [15]. (**B**,** F**) Visualization of the result from the proportional difference (PD) algorithm shows the absolute difference of the proportion of the grandpaternal alleles compared to that of grandmaternal alleles along chromosome 1 in offspring ID-256 (**B**) and chromosome 21 in offspring ID-258 (**F**). Five recombination positions were indicated by the local maxima for offspring ID-256, and one recombination position were indicated by the local maximum for offspring ID-258. (**C**,** G**) Visualization of the result from the cumulative continuity score (CCS) algorithm shows the CCS for the paternal and maternal chromosomes along chromosome 1 in offspring ID-256 (**C**) and chromosome 21 in offspring ID-258 (**G**). Five recombination positions in offspring ID-256 and one recombination position in offspring ID-258 were indicated (see maternal chromosome; border between orange and light blue at position ca. 3 Mb). (**D**,** H**) The local density of informative SNPs along chromosome 1 in offspring ID-256 (**D**) and chromosome 21 in offspring ID-258 (**H**)

The grandparent-of-origin (GoO) of all informative alleles of SNPs along the paternal and maternal chromosome 1 and 21 in offspring ID-256 and ID-258 were inferred by *RecView*. Here, we only present the results for chromosome 1 for offspring ID-256 and for chromosome 21 for offspring ID-258. A visual inspection of chromosome 1 in offspring ID-256 suggested three crossovers on the paternal chromosome and two on the maternal chromosome (Fig. 3A). For chromosome 21 in offspring ID-258, we observed one uncertain crossover at the beginning of the paternal chromosome (within the first 0.1 Mb) and one obvious crossover towards the middle of the maternal chromosome (Fig. 3E).

Both the PD and the CCS algorithms reported the five recombination events on chromosome 1 for offspring ID-256 (Fig. 3B and C; Table 1), and the obvious crossover on the maternal chromosome 21 for offspring ID-258 (Fig. 3F and G; Table 1). However, the uncertain crossover at the beginning of the paternal chromosome 21 for offspring ID-258 was not supported by either PD or CCS using default options (Fig. 3F and G; Table 1). The local density of informative alleles along the chromosome varies along the chromosomes (Fig. 3D and H) and for the six reported recombination positions the precision ranged between 216 and 1754 bp (Table 1).

**Table 1:** Putative recombination positions and precision for chromosome 1 in offspring ID-256 and for chromosome 21 in ID-258 based on the full dataset. Also given are the parental origin of the chromosome and the analysis algorithm (PD: proportional difference; CCS: cumulative continuity score)

Offspring	Chromosome	Origin	Algorithm	Scaffold & Orientation	Chromosomal position (bp)	Precision (bp)
ID-256	1	Paternal	PD	Contig4 +	1,675,083	216
ID-256	1	Paternal	PD	Contig4 +	30,646,553	775
ID-256	1	Paternal	PD	Contig108 -	117,669,883	223
ID-256	1	Maternal	PD	Contig4 +	1,496,864	268
ID-256	1	Maternal	PD	Contig4 +	36,938,880	1754
ID-256	1	Paternal	CCS	Contig4 +	1,674,925	216
ID-256	1	Paternal	CCS	Contig4 +	30,641,720	775
ID-256	1	Paternal	CCS	Contig108 -	117,669,374	223
ID-256	1	Maternal	CCS	Contig4 +	1,492,708	268
ID-256	1	Maternal	CCS	Contig4 +	36,936,350	1754
ID-258	21	Maternal	PD	Contig21 -	3,062,428	309
ID-258	21	Maternal	CCS	Contig21 -	3,059,096	309

### Downsampled datasets

For the 10%-downsampled dataset, the PD algorithm recovered only three of the five recombination positions previously detected using the full dataset on chromosome 1 in offspring ID-256; the recombination events at ca. 1.7 Mb on paternal chromosome and ca. 1.5 Mb on maternal chromosome were not reported (Table 2). In contrast, the CCS algorithm located all five recombination events on chromosome 1 previously detected by the full dataset in offspring ID-256. Regarding chromosome 21 in offspring ID-258, both PD and CCS algorithms located the crossover on the maternal chromosome previously reported with the full dataset (Table 2). As expected, the 10%-downsampled dataset showed decreasing resolution with lower estimated precision compared to the full dataset (1961–25,000 bp, Table 2).

The results of the 1%-downsampled datasets showed drastically reduced success in locating recombination positions (only 3 recombination events were reported with the CCS algorithm) and drastically lowered precision (100,000 bp; Table 2).

**Table 2:** Putative recombination positions and precision for chromosome 1 in offspring ID-256 and for chromosome 21 in ID-258 for the 10%- and 1%-downsampled datasets. Also given are the parental origin of the chromosome and the analysis algorithm (PD: proportional difference; CCS: cumulative continuity score)

Offspring	Chromosome	Origin	Algorithm	Scaffold & Orientation	Chromosomal position (bp)	Precision (bp)
*10% downsampled dataset*						
ID-256	1	Paternal	PD	Contig4 +	30,654,646	7692
ID-256	1	Paternal	PD	Contig108 -	117,671,695	2326
ID-256	1	Maternal	PD	Contig4 +	36,949,357	25,000
ID-256	1	Paternal	CCS	Contig4 +	1,675,140	1961
ID-256	1	Paternal	CCS	Contig4 +	30,636,962	7692
ID-256	1	Paternal	CCS	Contig108 -	117,758,436	3704
ID-256	1	Maternal	CCS	Contig4 +	1,517,974	2439
ID-256	1	Maternal	CCS	Contig4 +	36,941,588	25,000
ID-258	21	Maternal	PD	Contig21 -	3,063,137	2941
ID-258	21	Maternal	CCS	Contig21 -	2,888,890	5882
*1% downsampled dataset*						
ID-256	1	Paternal	CCS	Contig4 +	30,701,609	100,000
ID-256	1	Paternal	CCS	Contig108 -	117,668,229	20,000
ID-256	1	Maternal	CCS	Contig4 +	37,001,284	NA^a^
ID-258	21	Maternal	CCS	Contig21 -	3,051,932	50,000

^a^Precision cannot be calculated because there are no informative SNPs in the interval used for this calculation (division with zero)

## Discussion

Traditional methods for analysing recombination, such as linkage maps, LD and cytogenetics, provide broad-scale estimation of recombination rate variation along chromosomes and may allow detecting recombination hotspots [5–13]. Methods designed for these purposes have been further developed to handle the increasing volume of genotype data, and now incorporate new analytical techniques, such as machine learning and coalescent modelling, for inferring population-level recombination rates in genomes [27, 28]. However, these analyses do not pinpoint the precise chromosomal locations of individual recombination events. The emergence of next-generation sequencing and high-quality reference genomes enables localisation of specific recombination events using whole-genome genotype data of individuals in pedigrees. The principle is to identify boundary positions between chromosomal regions with distinct grandpaternal and grandmaternal origins [14, 29]. While some available software handling high-density genetic data in pedigrees allow localising recombination events, such as LepMap3 [30] and YAPP [31], there has been a lack of efficient software for analysing, outputting and visualising such data. *RecView* fills this gap by enabling detection and visualisation of recombination events with high-throughput sequencing data of three-generation pedigrees. It provides an interactive GUI for easy and flexible analysis execution, allowing the user to choose different parameter settings, preview results, use automated detection algorithms, and save plots and tables.

*RecView* determines the grandparent-of-origin (GoO) for each allele at every SNP in the offspring by leveraging the genotypes of all pedigree individuals. It constructs a genotype string for each SNP in the pedigree and infers the origin of each allele in the offspring by comparing the genotype string to a comprehensive dictionary of all possible GoO scenarios (Supplementary 1.2). This search-and-match process to infer GoO is highly computationally efficient because it utilises GoO inferences made *a priori* during the construction of the dictionary, thereby eliminating the need for executing a series of conditional processes for identical genotype strings.

*RecView* analyses all genotypes provided in the input file, including incorrect genotypes possibly caused by sequencing or mapping errors. Some of these incorrect SNPs will lead to genotype strings indicative of biological impossible segregation patterns across generations, and these SNPs are excluded from the output. Others may introduce noise in the data, resulting in conflicting GoO inferences compared to adjacent SNPs along the chromosome (e.g., several SNPs appear to be genotype errors at 22 Mb of paternal chromosome 1 in offspring ID-256, Fig. 3A). *RecView* does not filter out these erroneous genotypes because crossovers are often separated by large chromosome regions, permitting a certain level of acceptable noise in the data while still retaining the ability to locate recombination events. Both the PD and CCS methods can detect large-scale chromosomal regions segregating in the pedigree, regardless of such noise. However, as noise increases relative to SNP density, accurately inferring crossovers becomes more challenging. Erroneously called genotypes can complicate the detection of real recombination events, especially when the recombined region is small. This is typically less of an issue for crossovers, as recombination interference tends to separate crossover events [32]. However, it can be a serious problem when inferring gene conversion events (non-crossovers), which usually span only a few hundred base pairs [33, 34]. Reducing data noise can be achieved by conducting deeper sequencing and implementing stricter filtering criteria during the SNP calling phase prior to the *RecView* analyses.

*RecView* implements two algorithms, the PD (proportional difference) and the CCS (cumulative continuity score) algorithms, to accurately identify and locate recombination positions (Supplementary 1.3–1.4). The PD algorithm identifies positions where the two adjacent windows differ the most in terms of which grandparental alleles they capture. The user can specify the window size, with larger windows limiting the detection of small regions and recombination events near the chromosome ends, while smaller windows increase susceptibility to noise in the data. The CCS method identifies positions between two regions that contain at least a specified number of consecutive informative alleles from each grandparent. Compared to the PD algorithm and depending on the user-specified settings, the CCS algorithm could have a better potential to locate recombination events close to chromosome ends. However, it can be more sensitive to incorrectly called genotypes, as errors disrupt the continuity of informative alleles and may cause the CCS-value to fall below the specified threshold (see, e.g., region 0–3 Mb of chromosome 1 where frequent noise causes relatively short CCSs; regions shown in black in Fig. 3C). Considering these advantages and disadvantages of both algorithms, we recommend using both the PD and CCS methods when studying recombination events, particularly for species with a strong bias of recombination towards the telomeric ends of chromosomes. Additionally, it is advisable to test different parameter settings for specific study species and datasets. For example, reducing the window size in PD (i.e., lowering the radius parameter) from 550 (default) to 460 resulted in locating the two recombination events on chromosome 1 in offspring ID-256 that were missed with the default parameter for the 10%-downsampled dataset (see Table 2).

The resolution of the inferred recombination positions depends on the size of the recombined region and the distribution of informative SNPs. In regions with high density of informative SNPs, actual recombination positions are more likely to be located near informative alleles, resulting in higher resolution for the inferred recombination positions. Hence, there is a negative association between SNP density and recombination position resolution. To estimate the precision of putative recombination positions, we provide data of the reverse local density of informative alleles. This measure indicates the genomic size (in bp) covered by an informative SNP and varies across species (due to differences in heterozygosity) and sequencing techniques (due to differences in the number of called SNPs). Compared to the full dataset, the precision in the 10%- and 1%-downsampled datasets dropped 10- and 100-fold, respectively. It is important to note that several recombination positions were not detected in the downsampled data when using same settings on all datasets (as we did here; Table 2).

When analysing recombination, it is crucial to consider the completeness of the genome assembly as crossovers occurring in unassembled parts of genomes will go undetected. We strongly recommend a thorough evaluation of each analysed chromosome arm, which should have at least one obligate crossover, resulting in a 50% chance of a recombination event [35]. Chromosome arms with unusually few detected recombination events may indicate incompletely assembled regions of the genome. Similarly, inaccurately assembled chromosomes can lead to erroneous inferences of recombination numbers and positions. For example, if Contig108 on chromosome 1 had been incorrectly oriented in our great reed warbler assembly (+ instead of -), it would have resulted in the identification of an additional crossover event, leading to a small double-crossover event in the parental chromosome (see Fig. 3A). Consequently, an unintended application of *RecView* is its potential to aid in curating genome assemblies. If an analysis of multiple offspring consistently reveals putative recombination positions at the same specific location (especially if this coincides with scaffolds boundaries), it may indicate assembly errors that require correction.

We envision future development of *RecView* to incorporate genotype uncertainties, impute missing genotypes, perform unsupervised parameter optimisation, etc. Such improvements would likely facilitate analyses of chromosomes with a limited number of informative SNPs and low-coverage or reduced representation sequencing data.

## Conclusion

*RecView* provides a user-friendly GUI that facilitates identification of recombination positions using genome-wide data in three-generation pedigrees. We applied *RecView* on a great reed warbler pedigree to showcase its features. These include plotting the grandparent-of-origin (GoO) of informative alleles at SNPs along a chromosome, which enables easy detection of putative recombination positions. Additionally, we demonstrate how *RecView* employs two algorithms, the proportional difference (PD) and cumulative continuity score (CCS) algorithms, to locate putative recombination positions. Each algorithm has its strengths and weaknesses, and we recommend using both to ensure comprehensive identification of recombination positions. When simultaneously analysing multiple offspring and chromosomes, *RecView* produces result tables that list all putative recombination positions, along with their estimated precision based on the local density of informative alleles. Such data provide a valuable resource for studies seeking a comprehensive understanding of recombination patterns and processes. In summary, *RecView*’s intuitive GUI and algorithmic capabilities make it a valuable tool for researchers investigating specific recombination positions using genome-wide sequencing data of three-generation pedigrees.

## Availability and requirements

Project name: RecView.

Project home page: https://github.com/HKyleZhang/RecView.git.

Operating system(s): macOS, Linux.

Programming language: R language.

License: GPL-3.0 license.

Any restrictions to use by non-academics: licence needed.

### Supplementary Information

**Additional file 1.**

## Data Availability

*RecView* is available on GitHub (https://github.com/HKyleZhang/RecView.git). The datasets (the full-, 10%- and 1%-VCF files) and input files (the full-, 10%- and 1%-genotype files and the scaffold file) for the two selected chromosomes are available at Dryad (10.5061/dryad.2fqz612w5).

## Abbreviations

CCS: Cumulative continuity score
GoO: Grandparent-of-origin
GUI: Graphic user interface
LD: Linkage disequilibrium
PD: Proportional difference
SNP: Single nucleotide polymorphism
