# Supplementary material for: RecView: an interactive R application for locating recombination positions using pedigree data
Source: BMC Genomics. 2023 Nov 25;24:712. doi: 10.1186/s12864-023-09807-2 (PMC10676570; doi:10.1186/s12864-023-09807-2)
Supplement: Supplementary file 1 — Additional file 1. [file 12864_2023_9807_MOESM1_ESM.docx]

# Supplementary

## 1 Working details of *RecView*

### 1.1 Required genotype data from a three-generation pedigree

*RecView* requires genotype data of bi-allelic SNPs of the individuals in a three-generation pedigree that includes four grandparents (F0), two parents (F1), and at least one offspring (F2) (**Figure S1**). The analysis is conducted independently for each offspring. The recombination being analysed occurs in the F1s, involving genetic exchange between the homologous chromosomes inherited from the F0s. If the recombined chromatid is passed down to the F2, we can trace chromosomal regions to the (paternal or maternal) grandfather or the (paternal or maternal) grandmother. The genotype file with bi-allelic SNPs can be extracted from variant calling files using, *e.g.*, *VCFtools*.


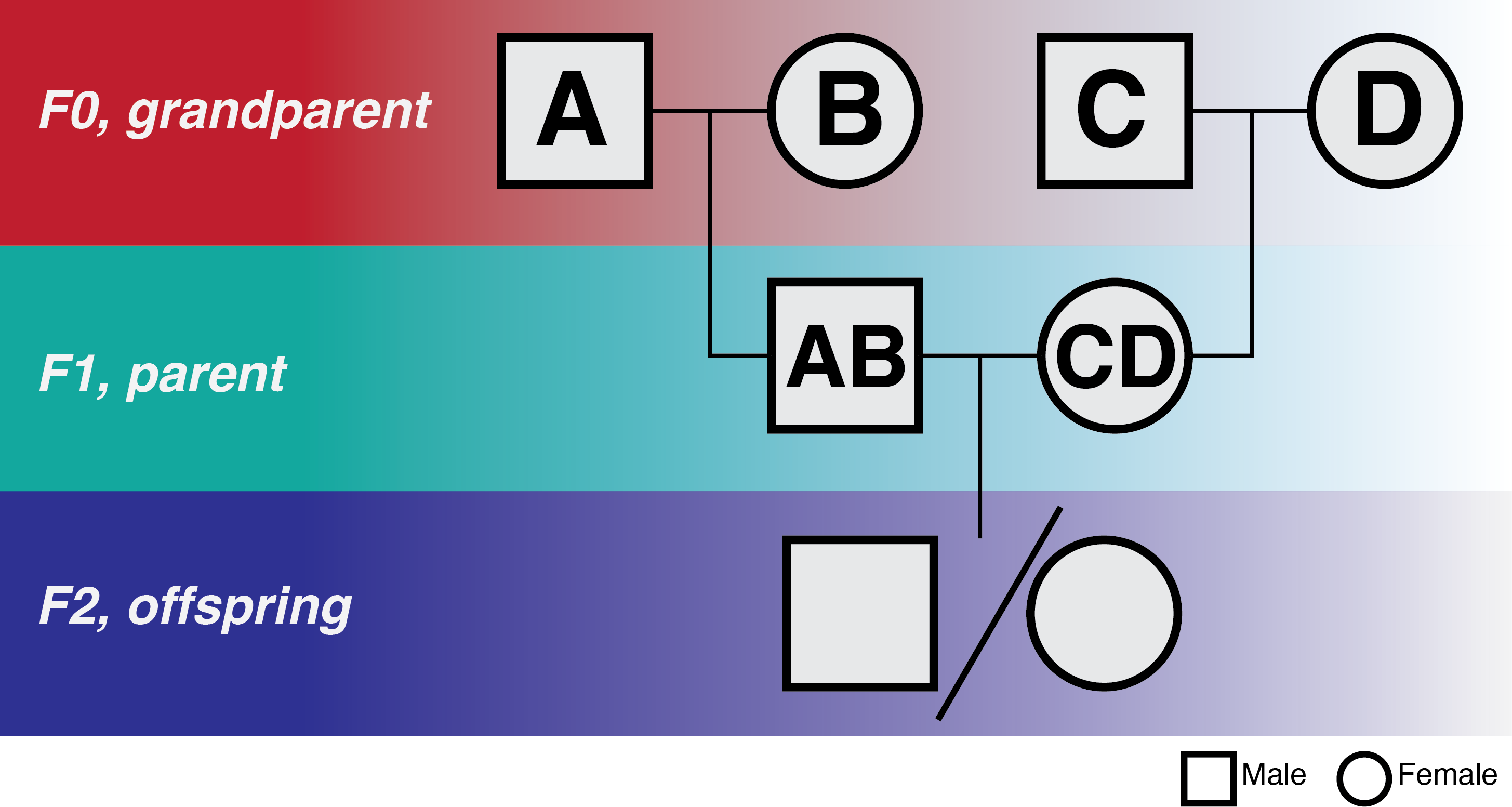


**Figure S1.** The pedigree dataset required for analysing recombination locations with *RecView.* Grandparents are labelled A, B, C and D, and parents AB and CD. The analysis is conducted independently for each offspring.

### 1.2 Dictionary of grandparent-of-origin (GoO)

*RecView* contains a “dictionary of GoO”, which includes all possible genotype strings of seven individuals in a three-generation pedigree. Technically, each genotype at each bi-allelic SNP is represented by 0, 1 or 2 where 0 = homozygote for the reference allele, 2 = homozygote for the alternate allele, and 1 = heterozygote. This means that a genotype string for grandparents A, B, C and D, parents AB and CD, and the offspring in Figure S1, can be written as 0-0-2-2-0-2-1 if both paternal grandparents are homozygous for the reference allele and both maternal grandparents are homozygous for the alternative allele, or 0-0-0-1-0-1-1 if the maternal grandmother, the mother and the offspring are heterozygous.

In total, there are 3^7^ = 2,187 different genotype strings, considering each individual has 3 possible genotypes (0, 1 or 2). We removed genotype strings that represent biologically impossible scenarios (ignoring mutations), for example, 0-0-0-0-1-0-1 where neither paternal grandparent carries the alternative allele present in the heterozygous father (and offspring). This leaves us with a dictionary of GoO with 435 biologically plausible genotype strings.

A specific dictionary of GoO is constructed for the X and Z chromosomes, or more precisely for the part of these chromosomes where the heterogametic sex does not recombine (XY males and ZW females). Basically, the dictionary of GoO is modified so that the GoO is not inferred for the paternal X chromosome in XY system and the maternal Z chromosome in ZW system.

As the dictionary of GoO contains all possible genotype strings and their corresponding inferred GoO, the 7-individual genotype string of each SNP can be used as keywords in the dictionary to obtain the GoO inference. The result can be visualised as the GoO of each SNP ordered by positions. **Figure S2** shows a hypothetical example of GoO of 200 SNPs along a small chromosome, which is also used for demonstrating the two algorithms to automatically locate recombination. *RecView* does not plot unresolved and thus uninformative alleles. Note that in this example, we use the order of the SNPs as their positions, which is not the case for real data where the actual positions on the chromosome are used for plotting and for interpretation of the actual recombination positions in the unit of base pair.

**
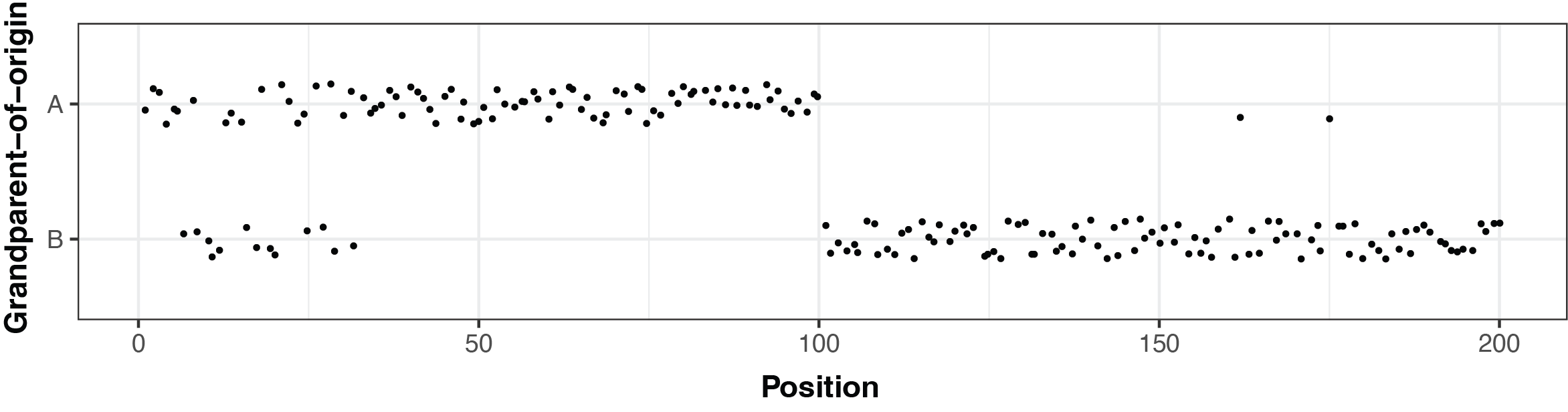
**

**Figure S2.** The paternal grandparent-of-origin inference for 200 SNPs. These data are hypothetical but were selected to indicate how, *e.g.*, sequencing errors may affect patterns. Data points are assigned with noise on the y-axis to avoid overlapping.

### 1.3 Proportional difference (PD) algorithm

The PD algorithm calculates the absolute difference in the proportion of alleles originating from a specific grandparent between flanking windows. It proceeds by specifying a window size (the number of informative SNPs of each flanking window), a step value (k) giving the number of SNPs between each calculated position, and a threshold to trigger denser calculations (*e.g.*, at every SNP) to detect local maxima.

Taking the example of the paternal chromosome, the evaluation at each position starts with calculating the proportion of alleles originating from grandparent A for the flanking windows (S1 for the downstream and S2 for upstream). Next, the absolute difference of the proportion A for S1 and S2 ($\left| \Delta_{GoO prop.} \right|$) is calculated for every k bp. If $\left| \Delta_{GoO prop.} \right|$ is smaller than the user-specified threshold, the process proceeds every k SNP, but if it is equal to or larger than the user-specified threshold, $\left| \Delta_{GoO prop.} \right|$ will be calculated for every informative SNP starting from the previous focal position (*i.e.*, current position - k). The step will return to a step size of k when $\left| \Delta_{GoO prop.} \right|$ is again smaller than the threshold. This design is intended to increase calculation speed while keeping the accuracy of the analysis. Putative recombination positions are defined as local maxima in $\left| \Delta_{GoO prop.} \right|$ above the threshold. **Figure S3** depicts the result of a PD analysis of the 200 SNPs in Figure 2 with settings of k = 5, window size = 19, and threshold = 0.85. The process of taking finer step starts from position 95 because position 100 is the first position (when using k = 5) with $\left| \Delta_{GoO prop.} \right|$ ≥ 0.85.

**
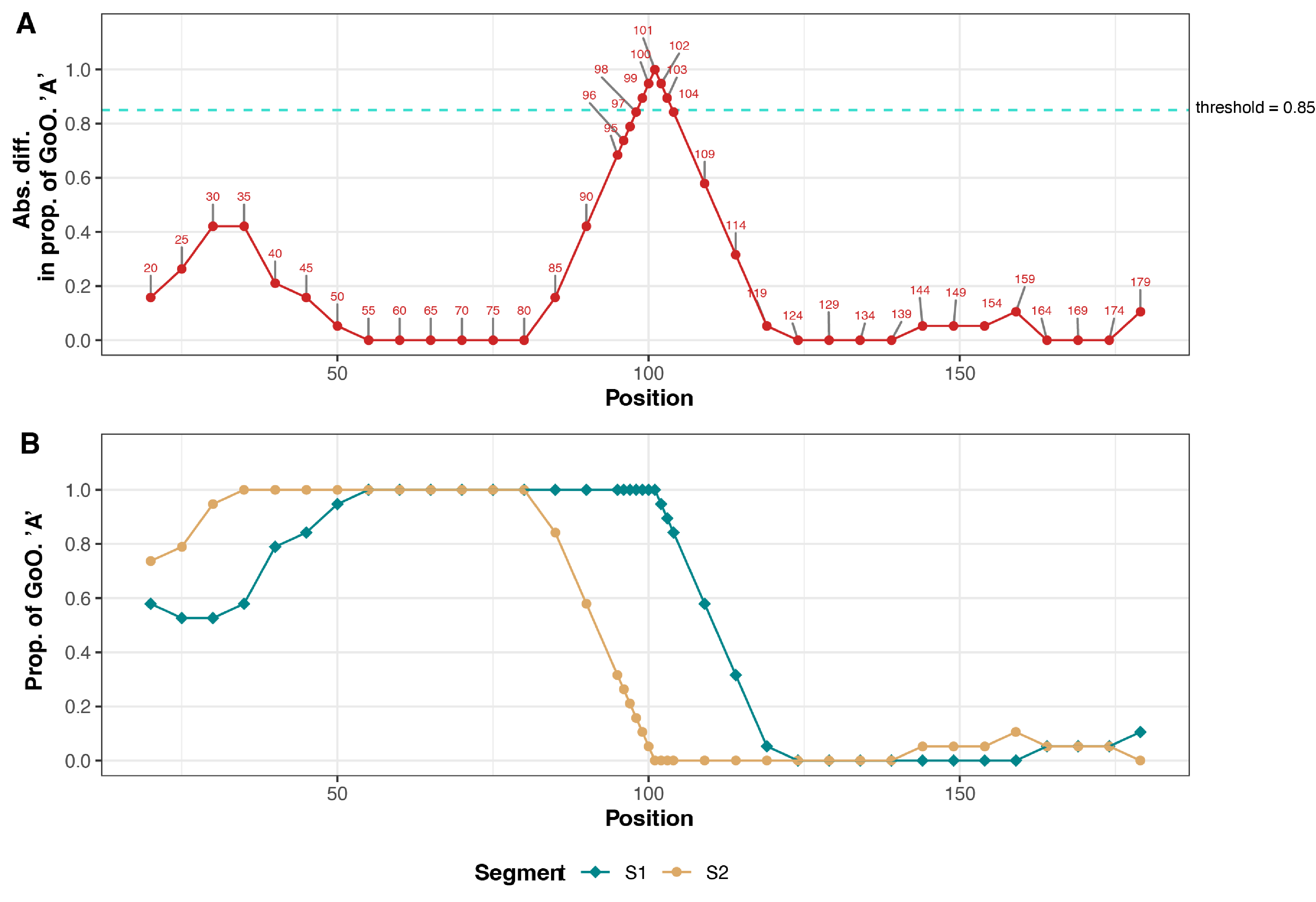
**

**Figure S3.** Illustrative demonstration of the proportional difference algorithm to automatically locate recombination positions. (**A**) The absolute difference of the proportion of the grandpaternal allele A for downstream (S1) and upstream (S2) windows $(\left| \Delta_{GoO prop.} \right|$) along the chromosome. (**B**) The proportion of the grandpaternal allele A for S1 (green) and S2 (yellow), respectively, at each focal position. Position 101 shows a local maximum above the threshold and is thus a putative recombination position.

### 1.4 Cumulative continuity score (CCS) algorithm

The CCS algorithm calculates a CCS for each position along the chromosome. The CCS describes the number of consecutively proceeding GoO inferences being the same as for the focal position (*e.g.*, CCS = 3 for the last position in BAAAA). The algorithm finds putative recombination positions by locating regions where long continuously increasing slopes of CCSs of one GoO is replaced by long continuously increasing slopes of CCSs from the other grandparent.

Taking the example of the paternal chromosome, if both the focal and the previous positions have GoO inference A, the focal position gets a continuity score of +1. Similarly, if the GoO inference is B for both the focal and the previous position, the focal position gets a continuity score of -1. In contrast, if the focal and the previous positions have different GoO inferences, the focal position will reset the CCS to 0. Plotting the CCS of each position along a chromosome will create increasingly positive and increasingly negative slopes (Figure 4) in which positive slopes indicate that the informative alleles in this region originate from the grandfather, whereas negative slopes indicate alleles originating from the grandmother.

To locate recombination positions, a user-specified threshold is used to exclude noise from mapping errors and wrongly called genotypes (which will reset CCS to 0). The selected threshold will depend on assumptions of the minimal size of the recombined regions, the density of the informative alleles, and the amount of noise in the data. **Figure S4** depicts the output of the 200 SNPs in **Figure S2** with a CCS threshold = 30, which implies that putative recombination positions will be located between the end of a positive (or negative) slope reaching above CCS = 30 and the beginning of the next negative (or positive) slope reaching above CCS = 30. We refer to these two positions as the left and right observed boundaries. In addition, note that slopes with values below the threshold might occur between these slopes (which is not the case in this example). In all cases, the putative recombination positions will be assigned the middle position between the left and right observed boundaries. In the example shown in **Figure S4**, a putative recombination position occurs between positions 100 and 101.


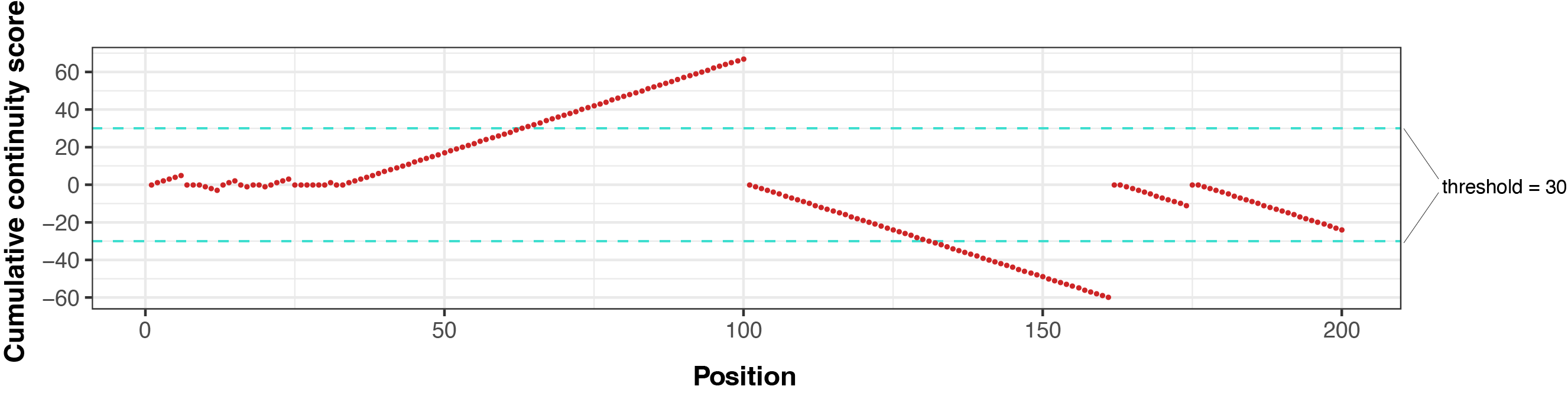


**Figure S4.** Illustrative demonstration of the cumulative continuity score (CCS) algorithm to automatically locate putative recombination positions. The CCS is reset to zero whenever the next grandparent-of-origin (GoO) inference is different. Positive and negative CCSs indicate continuity of GoO A or B, respectively. With a threshold of CCS = 30, there is one putative recombination position, between positions 100 and 101.

### 1.5 Estimated precision of putative recombination positions

To estimate the precision of putative recombination positions in different parts of the chromosome, we calculate the number of informative alleles in non-overlapping sliding windows of 100 Kb. The estimated precision is calculated as the reverse local density of informative alleles (*i.e.*, 1/local density of informative alleles), *i.e.*, the average number of base pairs per informative allele (unit: bp). Putative recombination positions located in a region with low local density have higher estimated error and lower certainty than those located in a region with high local density.
